# Circulating microRNAs as biomarkers for ischemic heart disease: a systematic review and gene set enrichment analysis

**DOI:** 10.3389/fmed.2025.1545023

**Published:** 2025-08-22

**Authors:** Yasmine Alcibahy, Radwan Darwish, Ghena Abu-Sharia, Quinten Maes, Omar Elgamassy

**Affiliations:** ^1^Royal College of Surgeons in Ireland – Bahrain, Al-Muharraq, Bahrain; ^2^College of Medicine, Alfaisal University, Riyadh, Saudi Arabia; ^3^School of Medicine, University of Groningen, Groningen, Netherlands

**Keywords:** ischemic heart disease (IHD), microRNAs, coronary artery disease (CAD), biomarkers, systematic review

## Abstract

**Introduction:**

Ischemic heart disease (IHD) remains a major global health burden, highlighting the urgent need for early, non-invasive diagnostic biomarkers. MicroRNAs (miRNAs), small non-coding RNA molecules that regulate gene expression, have emerged as promising candidates due to their stability in circulation and involvement in cardiovascular processes. This systematic review aimed to evaluate the potential of specific miRNAs as early diagnostic biomarkers in IHD.

**Methods:**

We conducted a systematic review following the Preferred Reporting Items for Systematic Reviews and Meta-Analyses (PRISMA) guidelines. Searches were performed in PubMed, Scopus, and Web of Science databases up to June 31, 2024. Eligible studies were selected based on predefined inclusion criteria. We identified recurrently dysregulated miRNAs and used miRTarBase to retrieve experimentally validated gene targets. Subsequently, gene set enrichment analysis (GSEA) was performed using Enrichr, referencing BioPlanet, KEGG, and Panther pathway libraries. Functional annotation was further explored using TAM 2.0.

**Results:**

A total of 38 studies met the inclusion criteria. Among the reported miRNAs, miR-126, miR-21, miR-145, miR-92a, and miR-155 were the most frequently and consistently dysregulated across various IHD subtypes. Although expression patterns varied, these miRNAs were recurrently implicated in IHD-related processes. GSEA revealed enrichment of their gene targets in key pathways such as p53, TGF-β, and FoxO signaling, as well as in processes involving apoptosis and angiogenesis critical in vascular injury, remodeling, and immune activation. Several cancer-related pathways were also enriched, underscoring molecular overlaps between tumorigenesis and atherosclerosis. TAM 2.0 functional annotation supported these findings, linking the selected miRNAs to smooth muscle differentiation, cytokine signaling, and regulation by key transcription factors including SMAD4, STAT3, and AP-1.

**Discussion:**

Our findings suggest that a panel combining the identified miRNAs may offer greater diagnostic value for IHD than individual markers, given their involvement in multiple IHD-related biological processes and pathways. The recurrent dysregulation of these miRNAs across diverse studies supports their potential as components of a robust, non-invasive diagnostic tool. However, expression variability and pathway overlap with other diseases, such as cancer, indicate the need for further validation. Larger prospective studies are warranted to validate their clinical applicability in IHD screening and risk stratification.

**Systematic review registration:**

International Prospective Register of Systematic Reviews (PROSPERO), identifier CRD42024583265.

## 1 Introduction

Ischemic heart disease (IHD) remains the world’s leading cause of mortality in countries of all income levels. Despite significant efforts to tackle this problem, which mainly affects the elderly, ineffective interventions and initiatives have only caused a minor decrease in mortality rates over the past decade (1). IHD most commonly develops as a result of atherosclerotic narrowing of the coronary artery lumen which leads to a lack of blood flow and ischemia of the heart’s muscle, known as coronary artery disease (CAD). The clinical presentation of CAD can vary significantly ranging from chronic stable angina to life-threatening acute myocardial infarction (AMI). Many risk factors are associated with IHD such as hypertension, hypercholesterolemia, diabetes mellitus, and obesity (2).

Currently, IHD is diagnosed using various methods including clinical examination, exercise stress testing, electrocardiography, echocardiography, CT angiography, and the gold standard; diagnostic coronary angiography. In cases of AMI, elevated levels of troponin and CK-MB can support diagnosis when correlated with ischemic symptoms and ECG changes (3). However, these protein-based biomarkers rise only after tissue damage has occurred, limiting their utility for early detection (4). This delay has driven interest in identifying earlier, more stable biomarkers; particularly in the form of microRNAs (miRNAs), which are now being explored for their potential to detect disease before irreversible damage sets in.

MicroRNAs are single-stranded, small, non-coding RNAs which usually have an average length of 22 nucleotides. They regulate cellular functions and gene expression by inducing messenger RNA (mRNA) degradation and translational repression. Additionally, they play a role in many biological processes like apoptosis, cellular proliferation, and communication. Once secreted, they remain stable in circulation; resistant to degradation in blood plasma or serum, making them suitable candidates for use as clinical biomarkers (5).

Recent studies have demonstrated that specific miRNAs can contribute to cardiovascular disease pathophysiology (6). Importantly, the clinical relevance of miRNAs extends beyond their potential as diagnostic biomarkers. A growing body of evidence supports their therapeutic utility, giving rise to the concept of theranomiRNAs; a term referring to miRNAs that possess both diagnostic and therapeutic potential. In cardiovascular medicine, several miRNAs have been shown not only to reflect disease presence but also to contribute directly to disease progression or resolution. For instance, some miRNAs promote endothelial dysfunction, vascular inflammation, or cardiomyocyte apoptosis, while others exhibit protective effects by enhancing angiogenesis or reducing fibrosis (7, 8). Therapeutically, miRNA mimics or inhibitors (antagomirs) are now being explored to modulate these effects *in vivo* (9, 10). The present systematic review explores the diagnostic value of circulating miRNAs in IHD. Based on frequency and consistency across studies, we identified a core set of miRNAs for further analysis. In addition to summarizing their expression trends in the literature, we performed *in silico* enrichment analysis to better understand their biological roles, regulatory networks, and relevance to cardiovascular pathology.

## 2 Methods

The review was carried out following a predefined protocol in line with the Preferred Reporting Items for Systematic Reviews and Meta-analysis (PRISMA) guidelines (11). Our study methodology was registered at the International Prospective Register of Systematic Reviews (PROSPERO), with registration number CRD42024583265. A comprehensive search of all original research published before 31 June 2024, was conducted across three major databases, including PUBMED, Scopus, and Web of Science.

### 2.1 Eligibility criteria

The eligibility criteria for the study were as follows: the population of interest was adult patients with IHD, with pediatric populations being excluded. The intervention focused solely on epigenetic biomarkers of the miRNA type, excluding other types of biomarkers such as mRNA, histone demethylase (HDMTs), histone acetyltransferase (HAT), histone deacetylase (HDAC), as well as non-epigenetic diagnostics and epigenetic-based therapeutics. The comparator was the biomarker levels among healthy adult patients. The outcome was limited to biomarker levels in the serum, with levels in other bodily fluids excluded. Regarding study characteristics, the analysis included observational studies and randomized controlled trials (RCTs) if available, while excluding non-human studies, reviews, case reports, letters, editorials, and conference abstracts.

### 2.2 Screening

Using the search strings related to or describing IHD and miRNA in the online databases PubMed, Scopus, and Web of Science, a total of 1,615 scientific articles were identified. These were first checked for duplicates, both manually and with the help of the online software Covidence (12). Two independent reviewers (Y.A. and G.A.-S.) performed the initial screening of study titles and abstracts, based on the predefined inclusion and exclusion criteria. A third reviewer (R.D.) resolved conflicts and made a final decision for a select number of disputed articles. The remaining articles were sought for retrieval and availability. Two independent reviewers (R.D. and Q.M.) then performed full-text screening, based on the same predefined inclusion and exclusion criteria. A third, independent reviewer (G.A.-S.) resolved conflicts and made a final decision for a select number of disputed articles.

### 2.3 Data extraction

For each study, two independent reviewers (Y.A. and O.E.) extracted data regarding study characteristics, such as study design and population. Additionally, Y.A. and O.E. separately collected information on the various miRNA profiles listed in each study and this was then documented (see Supplementary Table 1).

### 2.4 Quality assessment

The methodological quality of all included studies was evaluated using the Quality Assessment of Diagnostic Accuracy Studies (QUADAS-2) tool (13), specifically designed to assess diagnostic accuracy. To determine the risk of bias and the studies’ clinical relevance, this tool covers four domains: patient selection, index test, reference standard, and flow and timing of the study. Each domain includes specific questions that assess bias and applicability, with responses rated as “high,” “low,” or “unclear.” Studies were deemed of good quality if they provided essential details, such as the method used to identify biomarkers and cut-off values required for diagnostic purposes. The QUADAS-2 tool was independently conducted by two reviewers (Q.M. and Y.A.) and a third reviewer (R.D.) resolved any conflicts.

### 2.5 Gene set enrichment analysis

After identifying our differentially expressed miRNAs, we used the MiRTarBase (14), a curated dataset of target genes for each miRNA, to identify all associated genes. These were then compiled into a list, totaling 3,825 genes, that was inserted into Enrichr (15–17). Accordingly, we explored enriched pathways in three databases, BioPlanet 2019, KEGG 2021 Human, and Panther 2016 to see what they would reveal about the pathophysiology of IHD. Then, functional and disease association enrichment of the selected miRNAs was conducted using TAM 2.0 (18). Overrepresentation analysis was performed against the full human miRNA background with a minimum category size of 2. Cancer-related terms were masked to highlight cardiovascular-specific relevance.

## 3 Results

### 3.1 Study selection

**FIGURE 1:**
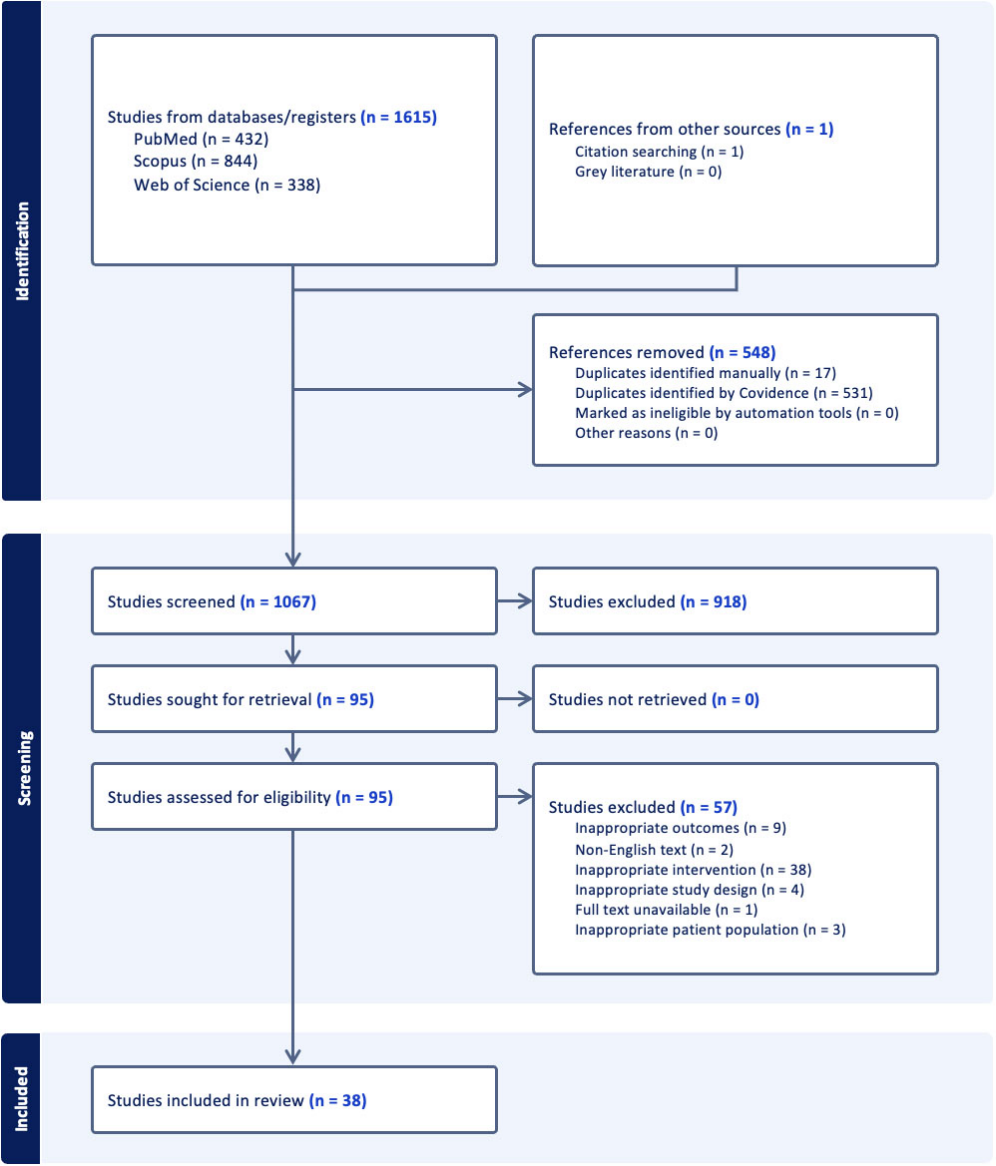
Flowchart illustrating the study selection process. Initially, 1,615 studies were identified from databases and registers, and 1 from other sources. After removing 548 duplicates, 1,067 studies were screened, with 918 excluded. Of the 95 sought for retrieval, none were unretrieved. Ninety-five were assessed for eligibility, with 57 excluded for reasons like inappropriate interventions and outcomes. Thirty-eight studies were included in the review.

In the initial database search, 1,615 studies were identified. After the removal of 548 duplicates, 1,067 records remained for title and abstract screening. During this phase, 918 studies were excluded based on the predefined eligibility criteria focusing on study population, intervention, outcomes, and study characteristics, resulting in 95 articles for full-text review. Of these, 57 studies were further excluded for various reasons. Nine were excluded based on inappropriate outcomes, in which miRNAs were mentioned but did form the focus of the study leading to outcomes that are not relevant to this systematic review. Two studies were excluded due to non-English text. Thirty-eight studies were excluded based on inappropriate intervention in which the intervention of the study did not sufficiently address the research question, and hence, data on miRNAs was lacking. Four were excluded due to inappropriate study design in accordance with the eligibility criteria. Furthermore, one study was excluded due to full text unavailability, and three studies were excluded based on inappropriate patient population also in accordance with the predefined eligibility criteria. Ultimately, 38 studies met the inclusion criteria and were selected for qualitative synthesis and included in the final systematic review. The process of study selection in the review is detailed in a PRISMA flowchart (Figure 1).

Based on these 38 studies, the five miRNAs miR-126, miR-21, miR-145, miR-92a, and miR-155 were selected based on their frequency of investigation, consistency of reported dysregulation, and known relevance to the pathophysiology of IHD. Among the 38 eligible studies, miR-126 was the most frequently reported, appearing in 19 studies, followed by miR-21 in 13 studies, miR-145 in 10 studies, miR-92a in 6 studies, and miR-155 in 8 studies. While some studies assessed multiple miRNAs, these five candidates emerged as the most consistently explored across diverse IHD subtypes. Their prevalence across studies supports their inclusion as the focus of this review and reflects both clinical and mechanistic interest in their diagnostic and prognostic utility.

Limiting the scope to five miRNAs also allowed for a more focused and integrative downstream *in silico* analysis, including target gene validation, pathway enrichment, and functional annotation. Expanding the list to include all miRNAs reported in the literature would have diluted the depth of the bioinformatic interpretation and introduced significant heterogeneity, given the inconsistent reporting standards, lack of replication, and variable diagnostic utility of many less frequently studied miRNAs. Therefore, the selection of these five candidates reflects a deliberate effort to prioritize robustness, reproducibility, and interpretability in both the evidence synthesis and subsequent biological modeling.

### 3.2 Study characteristics

The basic characteristics of the research articles which met the inclusion criteria and were selected for the systematic review are displayed in Supplementary Table 2 (3, 19–54). This includes the author names, year of publication, country, study design, sample sizes, and diagnostic criteria for IHD. Included articles were published between 2010 and 2024.

Note, many of the diagnoses were based around the guidelines published by the American College of Cardiology and American Heart Association (ACC/AHA). This refers to a set of guidelines meant to standardize and facilitate the diagnosis and treatment of cardiovascular conditions, like CAD. Furthermore, it helps in predicting a prognosis based on the patient risk factors and diagnostic results.

The current diagnostic criteria for CAD show a lack of focus when assessing the blood for certain miRNA biomarkers. Instead, the use of coronary angiography is favored which already has been researched more extensively and is considered the gold standard. When >50% of the diameter of at least one major coronary artery is obstructed, a diagnosis of CAD can be made. Additional measures like clinical examination, ECG, and echocardiography can further help distinguish between STEMI’s and NSTEMI’s as well as un-stable and stable angina pectoris.

### 3.3 Risk of bias assessment using QUADAS-2

**FIGURE 2:**
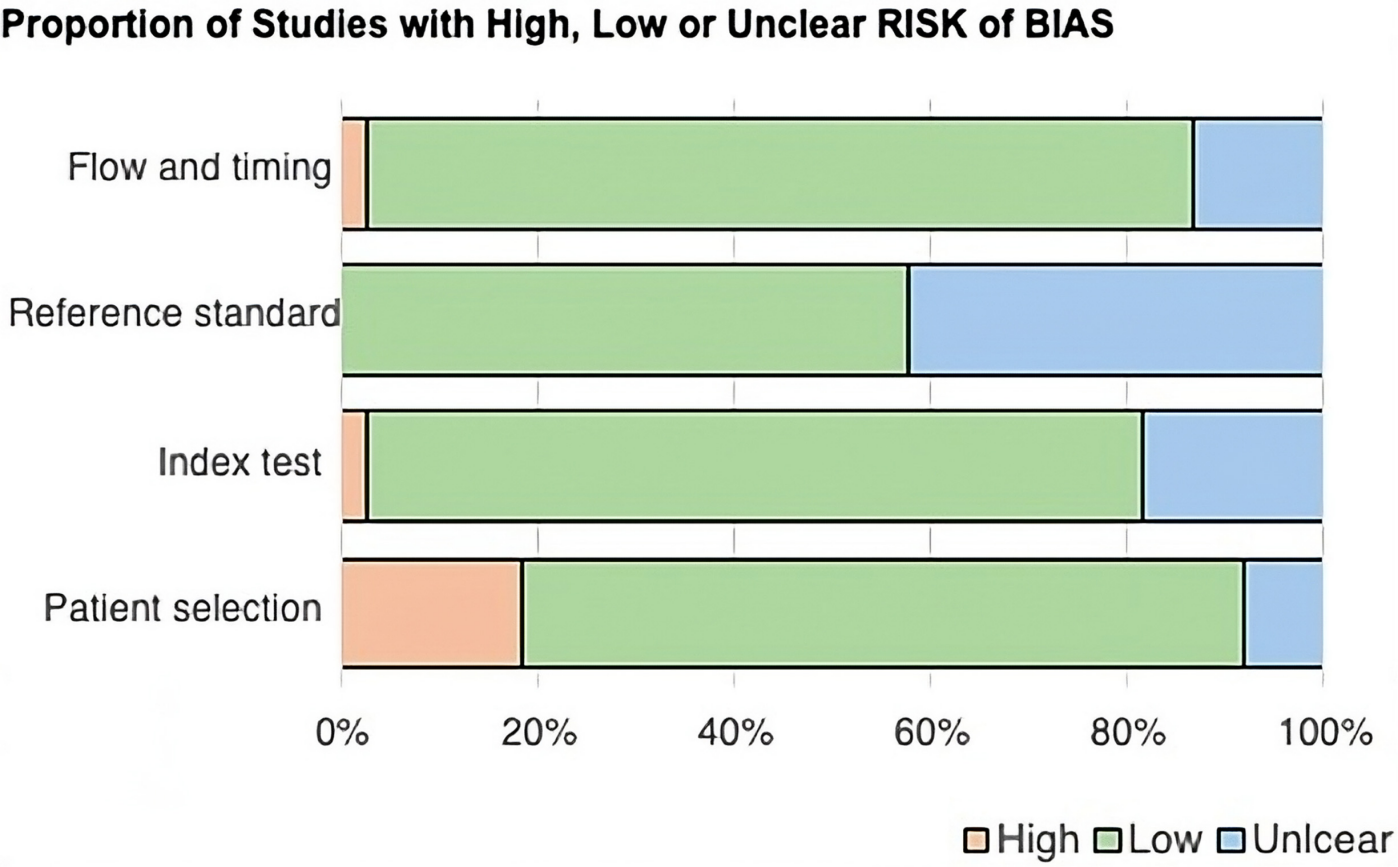
Bar chart showing the proportion of studies with high, low, or unclear risk of bias across four QUADAS-2 domains: flow and timing, reference standard, index test, and patient selection. Most domains have a low risk of bias, shown in green. Reference standard has more unclear risk, shown in blue, while patient selection has a notable high risk, shown in peach.

**FIGURE 3:**
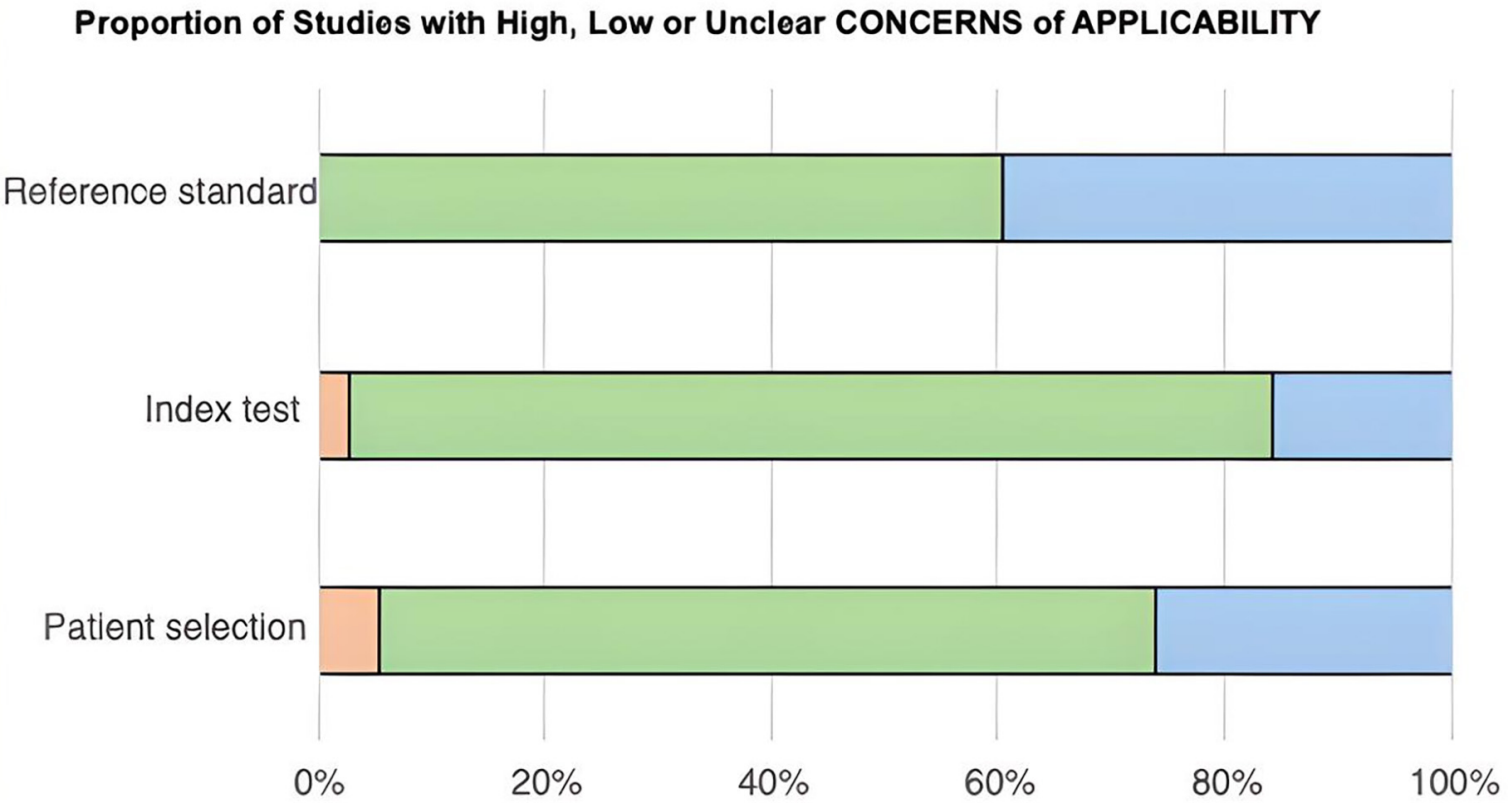
Bar chart showing the proportion of studies with high, low, or unclear concerns of applicability across three categories: reference standard, index test, and patient selection. Green indicates low concern, blue indicates unclear concern, and orange indicates high concern. Reference standard shows predominantly low concern. Index test has mostly low concern with some unclear. Patient selection has a mix of all three, predominantly low concern.

After the QUADAS-2 table was completed, two stacked bar graphs were created (Figures 2, 3), and the entire quality assessment is depicted in Supplementary Table 3. The first graph depicts the risk of bias focusing on four key domains including patient selection, index test, reference standard, and the flow and timing. The second graph displays the concerns related to the applicability which focuses on three key domains including the patient selection, index test, and reference standard. The majority of papers are seen to be low risk for both; however, a large proportion of papers have been identified as unclear, most prominently in the reference standard bar. In quantitative terms this is 42.11% and 40.54% for the risk of bias and concerns of applicability graphs, respectively. This can be explained by the fact that some papers solely compared the results from the index test to those from the control group, without comparing them to any predetermined threshold related to the reference standard. This is because some miRNAs have been less extensively researched in comparison to others, and hence, do not currently have any widely accepted thresholds to be used.

Additionally, the aims of some study were not specifically set out to determine the diagnostic accuracy regarding blood measurements of the respective miRNA, but rather to investigate whether they display significant upregulation or downregulation in respect to the control group. Therefore, a reference standard was often omitted from the paper as it did not serve as a requirement.

### 3.4 Data extracted from included studies

The following tables show a brief overview of the results from the 38 research articles that met all the selection criteria and from which the five miRNAs were selected for this systematic review. Note, some papers investigated multiple miRNAs, and hence, may be repeated. In most of the studies miRNAs were found to be either upregulated or downregulated when compared to the control group. The statistical tests were also analyzed to determine the significance of these results. In this systematic review, statistically significant findings are defined as a *P*-value ≤ 0.05. Lastly, additional analytical tools like the area under the curve (AUC), sensitivity, and specificity have been included, if they were mentioned, to further facilitate interpretation of the results and acknowledge diagnostic potential.

#### 3.4.1 miR-126 data extraction

Nineteen papers discussed miR-126 as part of their study (Table 1). Statistically significant results were shown in nine studies indicating upregulation (21, 27, 29, 31, 32, 35, 40, 55) and eight studies showing downregulation (3, 33, 37–39, 41, 43) of miR-126 levels for those who developed a heart problem related to IHD.

**TABLE 1:** Data extraction miR-126.

Authors	Validation method	Biomarker utility	miRNA profile	Statistical analysis	*P*-value
Zhong et al. (31)	qRT-PCR	1. UA vs. control 2. STEMI vs. controls	miR-126 ↑	1. AUC 0.714 2. AUC 0.703	*P* < 0.05
Masoodi Khabar et al. (32)	qRT-PCR	1. NSTEMI vs. STEMI 2. ACS vs. controls	1. miR-126 ↑ 2. miR-126 (n.s.)	Student’s *t*-test	1. *P* = 0.0272 2. *P* > 0.05
Wu and Zhang (33)	qRT-PCR	CAD vs. controls	miR-126 ↓	Student’s *t*-test AUC 0.801 Sensitivity 70.6% Specificity 85.4%	*P* < 0.001
Weber et al. (34)	qRT-PCR	CAD vs. controls	miR-126 remains unchanged	Student’s *t*-test	*P* > 0.05
Zhelankin et al. (21)	qRT-PCR	ACS vs. hypertension and controls	miR-126 ↑	Mann–Whitney *U* test	*P* < 0.05
D’Alessandra et al. (35)	qRT-PCR	UA and SA vs. controls	miR-126 ↑	AUC = 0.920	Not mentioned
Rizzacasa et al. (22)	qRT-PCR	CAD vs. AMI	miR-126 ↓	Student’s *t*-test, Mann–Whitney test	*P* < 0.05
Wang et al. (3)	qRT-PCR	CAD vs. controls	miR-126 ↓	Student’s *t*-test	*P* < 0.01
Reddy et al. (36)	qRT-PCR	CAD vs. controls	miR-126 (n.s.)	Student’s *t*-test, Mann–Whitney *U* test	*P* < 0.38
Ali et al. (37)	qRT-PCR	CAD vs. controls	miR-126 ↓	ROC curve analysis	*P* < 0.001
Zhang et al. (38)	qRT-PCR	CHD vs. controls	miR-126 ↓	Mann–Whitney test	*P* = 0.043
Ozuynuk-Ertugrul et al. (39)	qRT-PCR	1. MI vs. control 2. UAP vs. controls	miR-126 ↓	Kruskal–Wallis test	1. *P* = 0.008 2. *P* = 0.02
Taher et al. (40)	qRT-PCR	CAD vs. controls	miR-126 ↑	ROC-AUC = 0.976	*P* = 0.005
Su et al. (41)	qRT-PCR	CHD vs. controls	miR-126 ↓	Student’s *t*-test	*P* < 0.0001
Ekedi et al. (27)	qRT-PCR	TAA and CAD vs. controls	miR-126 ↑	ROC-AUC > 0.7	*P* = 0.000001
Xue et al. (42)	qRT-PCR	AMI vs. controls	miR-126 ↑	AUC = 0.72	*P* = 0.01
Fichtlscherer et al. (43)	qRT-PCR	Stable CAD vs. controls	miR-126 ↓	Student’s *t*-test, Mann–Whitney *U* test	*P* = 0.001
Ling et al. (29)	qRT-PCR	1. UA vs. controls 2. AMI vs. controls	miR-126 ↑	Mann–Whitney *U* test 1. AUC 0.781 2. AUC 0.849	1. *P* = 0.0005 2. *P* < 0.0001
Abdallah et al. (55)	qRT-PCR	CAD vs. controls	miR-126 ↑	Two-tailed Student’s *t*-test AUC 0.767	*P* < 0.001

qRT-PCR, quantitative reverse transcription polymerase chain reaction; CAD, coronary artery disease; miR-126, microribonucleic acid 126; n.s., not significant; SA, stable angina; UA, unstable angina; STEMI, ST-elevation myocardial infarction; NSTEMI, Non-ST-elevation myocardial infarction; AUC, area under the curve; ACS, acute coronary syndrome; AMI, acute myocardial infarction; ROC, receiver operating characteristic; CHD, coronary heart disease; TAA, thoracic aortic aneurysm; ↑, upregulated; ↓, downregulated.

#### 3.4.2 miR-21 data extraction

A total of 13 studies included miR-21 as part of their investigation (Table 2). Statistically significant findings were found in nine (20–24, 26, 27, 29, 55) studies indicating upregulation and two studies (25, 34) indicating downregulation of miR-21 in individuals suffering from a heart condition linked to IHD.

**TABLE 2:** Data extraction miR-21.

Authors	Validation method	Biomarker utility	miRNA profile	Statistical analysis
Dong et al. (19)	qRT-PCR	CAD vs. control	miR-21 (n.s.)	Student’s *t*-test
Samadishadlou et al. (20)	N/A	CAD and MI vs. control	miR-21 ↑	AUC 0.98 Sensitivity 0.91 Specificity 0.71
Zhelankin et al. (21)	qRT-PCR	CAD vs. control	miR-21 ↑	Mann–Whitney test
Rizzacasa et al. (22)	qRT-PCR	CAD vs. AMI	miR-21 ↑	Mann–Whitney *U* = 196
Darabi et al. (23)	qRT-PCR	ACS vs. stable CAD	miR-21 ↑	AUC 0.775 Sensitivity 0.74 Specificity 0.70
Kumar et al. (24)	qRT-PCR	CAD vs. controls	miR-21 ↑	AUC 0.79 Sensitivity and specificity both > 0.694
Telkoparan-Akillilar and Cevik (25)	qRT-PCR	CAD vs. control	miR-21 ↓	Student’s *t*-test
Ali Sheikh (26)	qRT-PCR	1. Stable angina vs. control 2. UAP vs. control	miR-21 ↑	1. AUC: SAP vs. healthy: 0.921 2. AUC: UAP vs. healthy: 0.944
Ekedi et al. (27)	qRT-PCR	TAA and stable CAD vs. controls	miR-21 ↑ in TAA	AUC > 0.8
Hortmann et al. (28)	ddPCR	CAD vs. control (at rest, stress, 2 h after stress)	miR-21 (n.s.)	AUC at rest = 0.51 AUC stress = 0.51 AUC 2 h after stress = 0.54
Ling et al. (29)	qRT-PCR	UA vs. AMI	miR-21 ↑	AUC 0.824
Abdallah et al. (55)	qRT-PCR	CAD vs. control	miR-21 ↑	AUC 0.767
Weber et al. (34)	qRT-PCR	CAD vs. control	miR-21 ↓	Student’s *t*-test

qRT-PCR, quantitative reverse transcription polymerase chain reaction; CAD, coronary artery disease; miR-21, microribonucleic acid 21; n.s., not significant; MI, myocardial infarction; AMI, acute myocardial infarction; AUC, area under the curve; ACS, acute coronary syndrome; SAP, stable angina pectoris; UAP, unstable angina pectoris; TAA, thoracic aortic aneurysm; UA, unstable angina; ↑, upregulated; ↓, downregulated.

#### 3.4.3 miR-145 data extraction

Ten papers were identified that looked at miR-145 levels as part of their study (Table 3). Statistically significant findings were seen in three studies showing upregulation (27, 42, 55), five studies showing downregulation (41, 43, 45, 49, 54), and one study showing no change in miR-145 levels (21) for those who developed a heart problem linked to IHD.

**TABLE 3:** Data extraction miR-145.

Authors	Validation method	Biomarker utility	miRNA profile	Statistical analysis	*P*-value
Du et al. (45)	qRT-PCR	CAD vs. controls	miR-145 ↓	Two-tailed Student’s *t*-test AUC 0.753 Sensitivity 0.675 Specificity 0.821	*P* < 0.001
Zhelankin et al. (21)	qRT-PCR	ACS vs. stable CAD vs. hypertension vs. controls	miR-145 remained unchanged	Mann–Whitney *U* test	*P* < 0.05
Gao et al. (49)	qRT-PCR	CAD and stable angina vs. controls	miR-145 ↓	Fisher’s test, Student’s *t*-test, Mann–Whitney *U* test	*P* < 0.001
Reddy et al. (36)	qRT-PCR	CAD vs. controls	miR-145 (n.s.)	Student’s *t*-test, Mann–Whitney *U* test	*P* < 0.08
Su et al. (41)	qRT-PCR	CHD vs. controls	miR-145 ↓	Student’s *t*-test	*P* < 0.0001
Ekedi et al. (27)	qRT-PCR	TAA and stable CAD vs. controls	miR-145 ↑	Mann–Whitney *U* test	*P* = 0.001
Xue et al. (42)	qRT-PCR	AMI vs. controls	miR-145 ↑	Student’s *t*-test, Mann–Whitney *U* test	*P* = 0.025
Fichtlscherer et al. (43)	qRT-PCR	Stable CAD vs. controls	miR-145 ↓	Mann–Whitney *U* test	*P* < 0.001
Abdallah et al. (55)	qRT-PCR	CAD vs. controls	miR-145 ↑	Two-tailed Student’s *t*-test AUC 0.959	*P* < 0.0001
Faccini et al. (54)	qRT-PCR	CAD vs. controls	miR-145 ↓	Student’s *t*-test, Mann–Whitney *U* test AUC 0.670	*P* = 0.003

qRT-PCR, quantitative reverse transcription polymerase chain reaction; CAD, coronary artery disease; miR-145, microribonucleic acid 145; n.s., not significant; AUC, area under the curve; ACS, acute coronary syndrome; CHD, coronary heart disease; TAA, thoracic aortic aneurysm; AMI, acute myocardial infarction; ↑, upregulated; ↓, downregulated.

#### 3.4.4 miR-92 data extraction

Nine studies included miR-92 in their paper (Table 4). Statistically significant findings were shown in three studies indicating upregulation (21, 48, 51) and two studies indicating downregulation (22, 43) in miR-92 levels when it comes to individuals with heart problems related to IHD.

**TABLE 4:** Data extraction miR-92.

Authors	Validation method	Biomarker utility	miRNA profile	Statistical test	*P*-value
Weber et al. (34)	qRT-PCR	CAD vs. control	miR-92 remains unchanged	Unpaired Student’s *t*-tests	*P* > 0.05
Liao et al. (48)	qRT-PCR	CHD vs. control	miR-92 ↑	Pearson correlation coefficient	*P* < 0.05
Rizzacasa et al. (22)	qRT-PCR	CAD vs. AMI	miR-92 ↓	Student’s *t*-test and Mann–Whitney test	*P* ≤ 0.05
Reddy et al. (36)	qRT-PCR	CAD vs. control	miR-92 expression levels were below detection limit in case and control.	Student’s *t*-test and Mann–Whitney *U* tests	*P* < 0.05
Fichtlscherer et al. (43)	qRT-PCR	Stable CAD vs. control	miR-92 ↓	Mann–Whitney test, ANOVA, Kruskal–Wallis test, and Student’s *t*-test	*P* < 0.05
Wang et al. (44)	qRT-PCR	CAD vs. control	miR-92 remains unchanged	Not mentioned	*P* < 0.05
Faccini et al. (54)	qRT-PCR	CAD vs. control	miR-92 (n.s.)	AUC = 0.520	*P* = 0.754
Zhelankin et al. (21)	qRT-PCR	ACS vs. stable CAD and hypertension ACS vs. control	miR-92 ↑ miR-92 (n.s.)	Mann–Whitney *U* test	*P* < 0.05 *P* = 0.1
Zhao et al. (51)	qRT-PCR	MI vs. control	miR-92 ↑	Paired sample *t*- test	*P* < 0.05

qRT-PCR, quantitative reverse transcription polymerase chain reaction; CAD, coronary artery disease; miR-92, microribonucleic acid 92; n.s., not significant; CHD, coronary heart disease; ANOVA, analysis of variance; AUC, area under the curve; ACS, acute coronary syndrome; MI, myocardial infarction; ↑, upregulated; ↓, downregulated.

#### 3.4.5 miR-155 data extraction

A total of eight papers were seen to discuss miR-155 (Table 5). Statistically significant results were identified in three papers showing upregulation (41, 50, 52) and five papers revealing downregulation (43, 46, 47, 54, 55) in miR-155 levels following the presence of a heart condition related to IHD.

**TABLE 5:** Data extraction miR-155.

Authors	Validation method	Biomarker utility	miRNA profile	Statistical analysis	*P*-value
Su et al. (41)	qRT-PCR	CHD vs. controls	miR-155 ↑	Student’s *t*-test AUC 0.846	*P* = 0.0018
Saadatian et al. (47)	qRT-PCR	Significant coronary stenosis (CAD–UAP, SAP, AMI) vs. insignificant coronary stenosis (ICAD) vs. controls	miR-155 ↓	Student’s *t*-test, Mann–Whitney *U* test AUC 0.74 Sensitivity 0.69 Specificity 0.79	*P* = 0.002
Zhu et al. (46)	qRT-PCR	CAD vs. controls	miR-155 ↓	Student’s *t*-test	*P* < 0.01
Qiu and Ma (52)	qRT-PCR	CHD vs. controls	miR-155 ↑	Student’s *t*-test	*P* < 0.001
Singh et al. (50)	qRT-PCR	CAD vs. controls	miR-155 ↑	Fisher’s test, Student’s *t*-test, Mann–Whitney *U* test	*P* < 0.05
Fichtlscherer et al. (43)	qRT-PCR	Stable CAD vs. controls	miR-155 ↓	Student’s *t*-test, Mann–Whitney *U* test	*P* < 0.001
Abdallah et al. (55)	qRT-PCR	CAD vs. controls	miR-155 ↓	Two-tailed Student’s *t*-test AUC 0.767	*P* < 0.001
Faccini et al. (54)	qRT-PCR	CAD vs. controls	miR-155 ↓	Student’s *t*-test, Mann–Whitney *U* test AUC 0.670	*P* = 0.04

qRT-PCR, quantitative reverse transcription polymerase chain reaction; CAD, coronary artery disease; miR-155, microribonucleic acid 155; CHD, coronary heart disease; AUC, area under the curve; UAP, unstable angina pectoris; SAP, stable angina pectoris; AMI, acute myocardial infarction; ↑, upregulated; ↓, downregulated.

### 3.5 Gene set enrichment analysis results

Given the wide range of biological processes implicated by the gene set shown in Supplementary Table 4, which we analyzed using *Enrichr*, it may be valuable to address these processes sequentially. This is shown in Table 6 below.

**TABLE 6:** Enriched pathways as defined by gene targets of miRNA-126, miRNA-21, miRNA-155, miRNA-145, and miRNA-92.

BioPlanet 2019	KEGG 2021 human	Panther 2016
Gene expression	Pathways in cancer	Angiogenesis
Pathways in cancer	Hepatitis B	CCKR signaling map ST
TGF-beta signaling	Proteoglycans in cancer	p53 pathway
P53 activity regulation	FoxO signaling pathway	p53 pathway feedback loops
Integrated breast cancer pathway	AGE-RAGE signaling pathway in diabetic complications	Apoptosis signaling pathway
T cell receptor regulation of apoptosis	Pancreatic cancer Salmonella infection	Ras pathway Insulin/IGF pathway-protein kinase B signaling cascade
Insulin signaling pathway	Cellular senescence	Oxidative stress response
ATM-dependent DNA damage response	Epstein-Barr virus infection	EGF receptor signaling pathway
Androgen receptor signaling, proteolysis, and transcription regulation	Hepatocellular carcinoma	FGF signaling pathway

Some of the commonly implicated pathways including p53 signaling (52, 53), apoptosis-related pathways (56, 57), TGF-β signaling (49), among others. Recent literature suggests that these pathways are associated with atherosclerotic plaque formation, vascular homeostasis, among other pathophysiological mechanisms. Further, TAM 2.0 enrichment analysis of the selected miRNAs (hsa-miR-126-3p, hsa-miR-21-5p, hsa-miR-145-5p, hsa-miR-92a-3p, and hsa-miR-155-5p) revealed a highly coordinated functional signature indicative of key biological processes underlying IHD. One of the most statistically significant enrichments was observed in smooth muscle cell (SMC) differentiation, driven by miR-21 and miR-145. This finding reflects the central role of vascular smooth muscle cell (VSMC) plasticity in atherosclerotic plaque development and vessel remodeling. While miR-145 is known to maintain the contractile phenotype of VSMCs and prevent their proliferation (56, 57), miR-21 promotes a shift toward a synthetic phenotype during vascular injury (58), implicating these miRNAs in both the progression and stabilization phases of atherosclerosis.

Another prominently enriched category was response to cytokine signaling, primarily through miR-126 and miR-155. This aligns with the known roles of these miRNAs in modulating vascular inflammation. miR-126 is typically associated with endothelial homeostasis and vascular repair, acting through repression of negative regulators like SPRED1 and VCAM1 (59, 60), while miR-155 is widely recognized as a pro-inflammatory miRNA that amplifies cytokine responses, particularly in macrophages and T cells (61, 62). Their enrichment suggests an important regulatory axis between endothelial dysfunction and immune cell activation in IHD. The category of intercellular communication was also significantly overrepresented, indicating that these miRNAs not only act cell-autonomously but also facilitate crosstalk between endothelial cells, immune cells, and vascular smooth muscle, a process increasingly recognized as central to plaque destabilization and thrombotic risk (63). Additional functional categories such as natural killer (NK) cell activation, apoptosis regulation, and radiation-induced bystander effects further clarify the broad but targeted immunovascular roles of this miRNA set. TAM 2.0 findings are summarized in Table 7 below.

**TABLE 7:** Summary of TAM 2.0 functional and disease enrichment for selected miRNAs.

miRNA	Enriched function(s)	Associated disease(s)	Key transcription factor(s)
miR-21-5p	Apoptosis, smooth muscle differentiation, cytokine response	Ischemia-reperfusion injury, cardiomyopathy, chronic atrial fibrillation, type 2 diabetes complications	AP-1, STAT3, SMAD3, SMAD4, KLF4
miR-126-3p	Endothelial cell regulation, cytokine signaling, intercellular communication	Atherosclerosis, type 2 diabetes complications, pneumonia, Sjogren’s syndrome	AP-1, TCF4, SMAD4
miR-145-5p	Smooth muscle cell differentiation, senescence	Peripheral vascular disease, postmenopausal osteoporosis, cardiovascular injury	SMAD4, BMP4, KLF2, IFNBETA
miR-92a-3p	Vascular injury, cell proliferation	Ischemia-reperfusion injury, vasculitis, lung injury	Not specified in TAM; known role in FoxO/PI3K pathways
miR-155-5p	Cytokine signaling, immune activation, NK cell function	Unstable angina, cardiomyopathy, autoimmune disease, Sjogren’s syndrome	AP-1, SMAD4, FOXP3, MYB, ETS2

## 4 Discussion

We reviewed 38 studies after applying our inclusion and exclusion criteria. Once we examined all the articles, we identified the most commonly recurring differentially expressed miRNAs from most to least frequently implicated: miRNA-126, miRNA-21, miRNA-145, miRNA-92, and miR-155. Gene targets were identified allowing us to perform a gene set enrichment analysis (GSEA).

In all studies, except the one conducted by Samadishadlou et al. (20), PCR tests were conducted to measure the expression levels of the respective miRNAs. Almost all of these used quantitative real-time PCR (qRT-PCR), however, one conducted by Hortmann et al. (28) opted for droplet digital PCR (ddPCR) instead. This form of PCR is more precise and sensitive when compared to qRT-PCR, and hence, will contribute toward strengthening the internal validity. However, it is more costly, complex, and time consuming (28).

### 4.1 miRNA-126

The literature indicated that miRNA-126 has a disease-dependent role, where the pattern of dysregulation is difficult to elucidate. However, miRNA-126 levels were upregulated in the studies that examined unstable IHD, ACS, UAP, STEMI, NSTEMI, or AMI patients (21, 27, 29, 31, 32, 35, 40, 42, 55). Levels were downregulated when stable IHD patients were examined (3, 22, 33, 34, 37–39, 41, 43).

This pattern suggests that miRNA-126 is associated with unstable plaque formation, which fits previous suggestions that it is associated with endothelial dysfunction, a hallmark of atherosclerosis (64). Accordingly, miRNA-126 can be used as a prognostic marker of IHD, as opposed to an early diagnostic one, as it indicates the severity of disease, and may help predict future cardiovascular events. These are speculations and cannot be answered conclusively without further studies.

### 4.2 miRNA-21

Overall, the levels of miRNA-21 in patients with IHDs compared to controls exhibited heterogeneity. In most studies, there was an upregulation of miRNA-21 in patients with IHD compared to controls, with a few discrepancies showing down regulation and some with no significant difference in the levels.

Of particular interest was the study conducted by Samadishadlou and colleagues (20), where miRNA-21 levels were compared between CAD, MI and control patients. It was concluded that MI patients had significantly (*P* < 0.05) higher miRNA-21 levels, when compared to CAD patients and controls, indicating that miRNA-21 can be a valuable differentiator between CAD and MI (20). In addition, this also suggests that miRNA-21 plays a vital role in different inflammatory and cardiovascular remodeling processes. In cases of cardiovascular disease, for instance, it can regulate the actions of peripheral blood mononuclear cells (PBMCs), responsible for reductions in Treg cells and accordingly reduce expression of TGF-β1, further promoting disease progression (65).

Further, the overexpression of miRNA-21 in IHD was seen to be associated with the apoptosis regulatory proteins [B-cell lymphoma 2 (BCL-2)] and phosphatase and tensin homolog (PTEN), suggesting an additional role as a regulator of the cell cycle in stress responses, as demonstrated in a study examining patients with stable, unstable angina and controls (26). Notably, the levels of miRNA-21 differed when measured at rest as opposed to 2 h after stress yield (28). At rest, the levels of miRNA-21 did not vary significantly between CAD patients and controls but were significantly greater in CAD patients when measured 2 h after stress yield. This, coupled with the fact that the mechanisms by which miRNA-21 functions are poorly understood, implies that the role of this miRNA is not as one-dimensional as it may seem, rather affected by different stages of the disease, different host factors, and a myriad of other extrinsic and intrinsic factors (28).

### 4.3 miRNA-145

As for miRNA-145, it has long been associated with cardiovascular physiology and pathology, implicated in the dysfunction of VSMCs, endothelial cells, fibroblasts and cardiomyocytes once vascular damage occurs (66). miRNA-145 levels were significantly reduced in STEMI patients compared to NSTEMI, stable angina and normal controls. Multivariable linear regression analysis was then used to demonstrate a marked association between miRNA-145 and increased vascular damage (49).

miRNA-145 levels were generally reduced in atherosclerotic conditions compared to hypertensive, stable CAD and healthy controls. It is thought that miRNA-145 has an atheroprotective function, protecting VSMCs from undergoing complete differentiation in the context of vascular disease (21, 66). Another study indicates that there is no significant difference in levels of miRNA-145 between different cohorts. However, a statistically significant inverse correlation was demonstrated between miRNA-145 levels and the total volume of fibroatheromas, with a particularly strong ability to predict the presence of thin cup fibroatheromas (27). Collectively, the evidence supports the atheroprotective role of miRNA-145 in cardiovascular disease.

### 4.4 miRNA-92

Further, no common relationship could be elucidated between the levels of miRNA-92 and the incidence of IHD patients. Regardless, valuable information regarding the function of miRNA-92 was gathered. It is an endothelial protein that is expressed to maintain vascular integrity, considering its association with the VEGF signaling pathway, rationalizing the downregulation of miRNA-92 among IHD patients in some included studies (43, 44). This opens new avenues of research for the use of miRNA-92 as a biomarker of endothelial and vascular integrity generally, as opposed to a diagnostic marker for a certain disease.

miRNA-92 levels were also significantly upregulated (*P* < 0.05) in cases of UAP compared to stable angina and controls, possibly suggesting the use of miRNA-92 as a differentiating biomarker, rather than a diagnostic one (48). Research on miRNA-92 allows us to argue that the wide variability seen in levels of miRNA when tested as biomarkers, is due to the specific functions it plays in different cardiovascular diseases. Seeing how often endothelial and vascular damage, transient or permanent, are implicated in cardiovascular pathology, the heterogeneity of results is expected.

### 4.5 miRNA-155

Historically aberrant expression of miRNA-155 has been associated with tumor progression and maintenance (67–69) and seeing the number of shared risk factors between malignancy and IHD, miRNA-155 was investigated for its role in IHD. Since then, it has been implicated as a key modulator of inflammatory processes. It holds atheroprotective functions, with results indicating that it is downregulated in patients with AMI and UAP (46).

Predominantly, miRNA-155 was found to be downregulated in patients with IHD when compared to controls. Additionally, the levels of miRNA-155 were lower within PBMCs in patients with IHD and happened to coincide with the severity of the coronary stenosis, as indicated by the Gensini scores. A significant negative correlation was observed in miRNA-155 levels and the Gensini scores (*P* < 0.001), indicating that lower levels of miRNA-155 are related to severe CADs. This is further supported by the marked associations between miRNA-155 and risk factors of IHD, including but not limited to cholesterol levels, hypertension, tobacco and age (46).

A similarly designed study found a positive correlation between the levels of miRNA-155 and Gensini scores (*r* = 0.6124, *P* < 0.001), demonstrating upregulation in IHD patients Perhaps miRNA-155 levels vary with different stages of the disease, but this cannot be ascertained with the current studies that did not differentiate between different stages and severity of IHD.

### 4.6 Gene set enrichment analysis

To better understand the role these miRNAs, play on a cellular level, we conducted a GSEA using BioPlanet, KEGG and Panther pathways on *Enrichr*. As a result, we were able to identify a wide variety of biological processes relevant to miRNAs and CAD.

In terms of cell cycle and apoptosis, the p53 pathway has been repeatedly implicated in our set of genes. Perhaps uncoincidentally, studies dating back to 1997 carried out by Wang et al. (70) have shown connections between single-nucleotide polymorphisms (SNPs) at p53, and increased predisposition to CAD. More recently, Zekavat et al. (71) have demonstrated that somatic mutations in blood mediated by the p53 gene lead to clonal hemopoiesis of indeterminate potential (CHIP), a preconceived normal aging process, and increase the risk of both CAD and PAD.

Apoptosis pathways are also implicated, where an excess has been shown to promote atherosclerotic processes (72), with the exception of VSMC-induced atherosclerosis (73), where data is yet controversial. Stalwart investigators were quick to realize that apoptosis levels are higher among patients who have had acute or alternatively sub-AMIs (74). This prompted further investigations on a molecular level that revealed that cleaved caspase-3 p17 peptide, an apoptosis-related cysteine peptidase, is not only elevated among CAD patients experiencing STEMIs, but also present within atherosclerotic plaques (75). Many more genes have been implicated in CAD, with BCL-2 rs17757541 C > G polymorphism showing close correlation with CAD in a Chinese population (76), p53, also an upstream regulator of Bax, accumulating in monocytes of CAD patients against a background of oxidative stress (77) (which has also been indicated by Panther 2016 database). Simvastatin, a well-known lipid-lowering drug known as a statin, significantly improves cardiac function among patients who have experienced AMI by, partially, increasing expression of BCL-2, and decreasing that of Caspase-3 and Bax (78).

This also brings us to the second part of the discussion, signaling pathways, which are, more often than not, associated with apoptosis. Most notably, pathways associated with members of the tumor necrosis factor (TNF) superfamily are of interest. Our analysis revealed that the TGF-β signaling pathway (BioPlanet 2019) is enriched, which is in agreeance with the role of TGF-β as a regulator of SMC and vascular homeostasis (79), or alternatively disease. Its role in CAD has been further solidified by genome-wide association studies (GWAS) that helped establish that SNPs in the promoter and coding region of TGF-β1 increase the risk of AMI (80, 81). Notably, our TAM 2.0 output also identified SMAD3 and SMAD4, downstream effectors of TGF-β signaling, as transcription factors enriched among miR-145 and miR-155 targets. This suggests that the miRNA panel may act upstream of TGF-β-driven transcriptional programs.

The data remains under close scrutiny, because experimental models indicate that TGF-β can be both atheroprotective and atherogenic. Earlier studies showed that global TGF-β inhibition or knockdown approaches collectively led to an increase in atherosclerotic plaque formation, and thrombotic risk (82). In support of these results, overexpression of TGF-β1 was shown to reduce pro-inflammatory plaque formation and oxidative stress, yet another pathway implicated in our analysis (83). These views are however complicated by recent findings that have demonstrated the atherogenic nature of TGF-β, driving the trans-differentiation of SMC into myofibroblasts, an integral step toward plaque formation. Also, perhaps uncoincidentally, the literature points to miRNA-21 as a negative regulator of Treg cells, mediated through a TGF-β1/Smad-independent pathway in patients with IHD (84, 85).

### 4.7 Future perspectives and clinical translation

In addition to their role as diagnostic biomarkers, miRNAs have demonstrated therapeutic potential in IHD. Due to their capacity to regulate gene expression post-transcriptionally, miRNAs are capable of modulating a range of complex pathophysiological processes, including endothelial dysfunction, inflammatory responses, foam cell formation, and fibrous cap destabilization (86–88). Therapeutic strategies have been developed to either restore the function of downregulated, cardioprotective miRNAs using synthetic mimics or inhibit the expression of pathogenic miRNAs using antagomirs (89). Notably, inhibition of miR-155 has been shown to reduce the formation of foam cells through regulation of CEH, ABCA1, and SR-A, thereby enhancing cholesterol efflux and decreasing lipid accumulation (86). miR-21 has been implicated in both promoting angiogenesis and contributing to plaque instability through the suppression of PTEN expression (90, 91). In contrast, overexpression of miR-145 has been associated with attenuation of endothelial injury and inflammation in patients with ACS, primarily through targeting of the FOXO1 gene (87).

The therapeutic efficacy of miRNA modulation has been further substantiated in several preclinical studies. In rodent models of AMI, administration of miR-21 and miR-214 has been associated with improvements in ventricular remodeling and reductions in cardiomyocyte apoptosis (92, 93). Similarly, in a porcine model, intracoronary delivery of anti-miR-92a was found to enhance angiogenesis and prevent adverse post-infarction remodeling (94). Despite these promising outcomes, significant barriers remain in the clinical translation of miRNA-based therapies. These include the risk of off-target effects, rapid enzymatic degradation, immune activation, and the challenge of achieving efficient and tissue-specific delivery (95).

To mitigate these limitations, several advanced delivery platforms have been developed. Liposomal nanoparticles have demonstrated success in delivering miR-153-3p to the myocardium, resulting in significant cardioprotective effects in rat models of myocardial infarction (96). In addition, stem cell-derived exosomes have emerged as a promising cell-free delivery vehicle, offering enhanced stability, low immunogenicity, and improved cellular uptake (97). Furthermore, utilizing miRNA profiling into clinical practice may facilitate the implementation of personalized therapeutic approaches, enabling stratification of patients based on disease phenotype and progression (89).

## 5 Conclusion

This systematic review identified the five most studied, consistently dysregulated miRNAs in IHD: miR-126, miR-21, miR-145, miR-92a, and miR-155. While these miRNAs repeatedly appeared across different studies, the direction of their expression varied. This inconsistency likely reflects the complex and context-dependent roles these miRNAs play in disease, rather than an error in methodology. GSEA tied the gene targets of these miRNAs to several relevant pathways; including p53, TGF-β, angiogenesis, and apoptosis, supporting their involvement in cardiovascular pathology. TAM 2.0 further connected them to SMC regulation, cytokine signaling, and key transcription factors like SMAD4 and STAT3.

However, these findings also revealed that miRNAs are not disease-specific by nature, rather influenced by disease, disease subtype, disease stage, patient background, and sample timing. This became particularly apparent for miR-126, which was upregulated in acute myocardial events, but downregulated in chronic disease.

That said, it may be more practical to approach miRNA diagnostics as a panel-based system, rather than relying on single markers. A multi-miRNA panel could better capture the complexity of IHD across different presentations. Future research should focus on comparing miRNA expression across defined IHD subtypes and disease phases, with consistent methodology, to clarify their diagnostic roles and potential clinical use.

## Data Availability

Publicly available datasets were analyzed in this study. This data can be found here: https://mirtarbase.cuhk.edu.cn/~miRTarBase/miRTarBase_2025/php/index.php, https://maayanlab.cloud/Enrichr/.
