# Supplementary material for: Circulating microRNAs as biomarkers for ischemic heart disease: a systematic review and gene set enrichment analysis
Source: Front Med (Lausanne). 2025 Aug 22;12:1545023. doi: 10.3389/fmed.2025.1545023 (PMC12411434; doi:10.3389/fmed.2025.1545023)
Supplement: Supplementary file 2 [file Table_2.docx]

**Table S1: Study Characteristics**

| **#** | **Authors** | **Year** | **Country** | **Study design; setting** | **Patient Characteristics** | | **Diagnosis** |
| --- | --- | --- | --- | --- | --- | --- | --- |
|  |  |  |  |  | **Control** | **Cases** |  |
| 1 | Dong et. al (1) | 2017 | Beijing, China | Observational;  hospital | 149 healthy controls. | 161 stable CAD patients | CAD confirmed by coronary angiography. |
| 2 | Samadishadlou et. al (2) | 2023 | Tabriz, Iran | Observational; based on datasets | 51 healthy controls. | 46 CAD, 111 MI patients. Total = 157 | Based on 3 gene expression datasets –N/A. |
| 3 | Zhelankin et. al (3) | 2021 | Moscow, Russia | Observational; cardiology clinic | 30 healthy controls. | 50 patients with ACS, 26 patients with stable CAD, 30 hypertensive patients without CAD. | Patients were diagnosed according to the European Society of Cardiology criteria. |
| 4 | Rizzacasa et. al (4) | 2019 | Rome, Italy | Observational; hospital | N/A. This study compared miRNA levels between stable and unstable CAD. | 61 patients with stable CAD.  38 patients had unstable CAD (AMI). Total = 99 | Coronary angiography. |
| 5 | Darabi et. al (5) | 2016 | Isfahan, Iran | Observational; hospital | N/A. | 50 patients with ACS.  50 patients with stable CAD. | Based on results of coronary angiography, ECG and clinical symptoms. |
| 6 | Kumar et. al (6) | 2020 | New Delhi, India | Observational; hospital | 54 healthy controls. | 78 patients with CAD. | Coronary angiography. Diagnosed according to the ACC/AHA 2007 guidelines. |
| 7 | Telkoparan-Akillilar et. al (7) | 2021 | Ankara, Turkey | Observational; cardiovascular clinics | 26 healthy controls. | 25 patients with SA. | Coronary angiography. |
| 8 | Ali Sheikh (8) | 2020 | Jouf, Saudi Arabia | Observational; hospital setting | 50 healthy controls. | 123 patients with SA.  82 patients with UA. | Stable and unstable coronary patients were confirmed following AHA/ACC clinical protocols. |
| 9 | Ekedi et al. (9) | 2023 | Moscow, Russia | Observational; hospital | 17 healthy controls. | 38 TAA patients.  67 patients with stable CAD. | Echocardiography, coronary angiography |
| 10 | Hortmann et al. (10) | 2019 | Basel, Switzerland | Observational; hospital | 101 healthy controls. | 99 patients with CAD. | MPI-SPECT/CT, coronary angiography, and fractional flow reserve |
| 11 | Ling et al. (11) | 2020 | Changchun, China | Observational; hospital | 22 healthy controls. | 34 patients with AMI.  31 patients with UA. | AMI was diagnosed in accordance with the European Society of Cardiology guidelines.  UA was diagnosed based on the patient history, presenting signs and symptoms, coronary angiography, and ECG. |
| 12 | Abdallah et al.(12) | 2022 | Ismailia, Egypt | Observational; cardiology clinic | 73 healthy controls. | 73 patients with CAD. | CAD diagnosed by clinical examination and diagnostic tools (Echo and ECG). |
| 13 | Zhong et al. (13) | 2018 | Guangdong, China | Observational; hospital | 26 healthy controls. | 26 patients with unstable angina.  26 patients with STEMI. | Laboratory tests, ECG and coronary angiography (according to the European Society of Cardiology/American College of Cardiology). |
| 14 | Khabar et al. (14) | 2023 | Tehran, Iran | Observational (case-control); hospital | 13 healthy controls. | 40 patients with CAD of which 23 were further diagnosed with ACS and 17 with SA. | Diagnosis of ACS and classification into STEMI, NSTEMI, and UA was done using the international ACS diagnostic criteria. |
| 15 | Wu et al. (15) | 2018 | Wuhan, China | Observational (case-control) | 96 healthy controls. | 19 patients with CAD. | Coronary angiography |
| 16 | Weber et al. (16) | 2011 | Atlanta, GA, USA | Observational (case-control) | 5 healthy controls provided blood for microarray analysis on RNA.  15 healthy controls provided blood for qRT-PCR analysis of miRNA expression. | 5 male subjects with CAD provided blood for microarray analysis on RNA.  10 patients with CAD provided blood for qRT-PCR analysis of miRNA expression. | Coronary angiography following AHA/ACC classification system. |
| 17 | Abdallah et al. (17) | 2023 | Ismailia, Egypt | Observational (case-control); cardiology clinic. | 90 healthy controls. | 90 patients with CAD. | Clinical examination, ECG, and echocardiography. |
| 18 | D'Alessandra et al. (18) | 2013 | Milano, Italy | Observational (case-control) | 20 healthy controls. | 53 patients with CAD. | Quantitative coronary angiography following ACC/AHA guidelines. |
| 19 | Wang et al. (19) | 2017 | Changzhi, China | Observational (case-control); hospital | 25 healthy controls. | 60 patients with CAD. | Coronary angiography was used to confirm CAD.  Ischaemic symptoms, increased levels of troponin and creatine kinase, ST-segment anomalies, and pathological Q-wave for AMI. |
| 20 | Reddy et al. (20) | 2019 | Mumbai, India | Observational; hospital | 31 controls (less than 30% stenosis). | 47 patients with CAD. | All patients underwent coronary angiography. |
| 21 | Ali et al. (21) | 2021 | Lucknow, India | Observational (case-control) | 100 healthy controls. | 100 patients with CAD. | Coronary angiography using ACC/AHA classification. |
| 22 | Zhang et al. (22) | 2018 | Xingtai, China | Observational (case-control); hospital | 20 healthy controls in exploration stage.  92 healthy controls in validation stage. | 20 patients with CHD in exploration stage. 102 patients with CHD in validation stage. | Coronary angiography. |
| 23 | Ozuynuk-Ertugrul et al. (23) | 2024 | Ankara, Turkey | Observational; hospital | 55 healthy controls. | 48 patients with SA. 46 patients with UA. | Coronary angiography  (lack of information) |
| 24 | Taher et el. (24) | 2023 | Baghdad, Iraq | Observational (case-control); hospital | 40 healthy controls. | 8 patients with CAD. | All patients were clinically diagnosed. |
| 25 | Su et al. (25) | 2020 | Heilongjiang, China | Observational (case-control); hospital | 126 high-risk controls and 18 healthy controls. | 203 patients with CHD | Coronary angiography (>50% luminal narrowing). |
| 26 | Xue et al. (26) | 2019 | Qingdao, China | Observational (case-control); hospital | 21 healthy controls. | 29 patients with AMI (15 STEMI and 14 NSTEMI) | AMI criteria* |
| 27 | Fichtlscherer et al. (27) | 2010 | Frankfurt, Germany | Observational (case-control) | 14 healthy controls. | 31 patients with stable CAD. | Coronary angiography. |
| 28 | Wang et al. (28) | 2020 | Beijing, China | Observational (case-control); hospital | 2 populations:  1) 39 healthy controls from Xuanwu Hospital  2) 21 healthy controls from Hospital of Yanbian University | 2 populations:  1) 39 patients with CAD from Xuanwu Hospital  2) 30 patients with CAD from Hospital of Yanbian University | Coronary angiography (>50% luminal narrowing). |
| 29 | Du et al. (29) | 2016 | Beijing, China | Observational (case-control); hospital | 60 healthy controls. | 60 patients with stable CAD. | Coronary angiography (>50% luminal narrowing). |
| 30 | Zhu et al. (30) | 2014 | Chongqing, China | Observational (case-control); hospital | 54 healthy controls. | 56 patients with CAD. | Coronary angiography using the Gensini score. |
| 31 | Saadatian et al. (31) | 2023 | Tehran, Iran | Observational (case-control); hospital | 74 healthy controls. | 102 patients with CAD. | Coronary angiography (>50% luminal narrowing). |
| 32 | Liao et al. (32) | 2019 | Beijing, China | Observational (case-control); hospital | 20 healthy controls. | 40 patients with unstable CAD | Coronary angiography (>50% luminal narrowing) which also met the ACC/AHA criteria. |
| 33 | Gao et al. (33) | 2015 | Beijing, China | Observational; hospital | N/A | 167 patients diagnosed with CAD. | Coronary angiography (>50% luminal narrowing) which also met the ACC/AHA criteria. |
| 34 | Singh et al. (34) | 2020 | Amsterdam, The Netherlands | Observational (case-control); hospital | 1^st^ validation cohort:  - 192 healthy controls  2^nd^ validation cohort:  - 250 healthy controls | **1^st^ validation cohort:**  - 64 patients with unstable CAD.  - 139 patients with stable CAD.  **2^nd^ validation cohort:**  - 250 patients with unstable CAD.  - 250 patients with stable CAD. | Coronary angiography. |
| 35 | Zhao et al. (35) | 2019 | Taiyuan, China | Observational (case-control); hospital | 14 healthy controls. | 14 patients with MI and 10 with SA. | Coronary angiography, ECG, and cardiac troponin tests. |
| 36 | Qiu et al. (36) | 2018 | Wenzhou, China | Observational (case-control); hospital | 100 healthy controls. | 300 patients with CHD. | Coronary angiography (>50% luminal narrowing). |
| 37 | Maciejak et al.(37) | 2016 | Warsaw, Poland | Observational (case-control) | 7 healthy controls. | 30 patients with CAD (STEMI) | All patients with CAD were diagnosed as a part of a previous study. |
| 38 | Faccini et al. (38) | 2017 | Toulouse, France | Observational; not stated. | 32 healthy controls. | 69 patients with CAD. | Not mentioned how CAD was diagnosed initially. |

CAD = Coronary Artery Disease; MI = Myocardial Infarction, AMI = Acute Myocardial Infarction; ACS = Acute Coronary Syndrome; ECG = Electrocardiography; ACC/AHA = American College of Cardiology / American Heart Association; SA = Stable Angina; UA = Unstable Angina; TAA = Thoracic Aortic Aneurysm; MPI-SPECT/CT = Myocardial Perfusion Single-Photon Emission Tomography / Computer Tomography; STEMI = ST-Elevation Myocardial Infarction; NSTEMI = Non-ST-Elevation Myocardial Infarction; CHD = Coronary Heart Disease; CPS = Chest Pain Syndrome; miRNA = Microribonucleic Acid; qRT-PCR = Quantitative Reverse Transcription Polymerase Chain Reaction
